# Electronic and chemical structure of the H_2_O/GaN(0001) interface under ambient conditions

**DOI:** 10.1038/srep24848

**Published:** 2016-04-25

**Authors:** Xueqiang Zhang, Sylwia Ptasinska

**Affiliations:** 1 Radiation Laboratory, University of Notre Dame, Notre Dame, IN 46556, USA; 2 Department of Chemistry and Biochemistry, University of Notre Dame, Notre Dame, IN 46556, USA; 3 Department of Physics, University of Notre Dame, Notre Dame, IN 46556, USA

## Abstract

We employed ambient pressure X-ray photoelectron spectroscopy to investigate the electronic and chemical properties of the H_2_O/GaN(0001) interface under elevated pressures and/or temperatures. A pristine GaN(0001) surface exhibited upward band bending, which was partially flattened when exposed to H_2_O at room temperature. However, the GaN surface work function was slightly reduced due to the adsorption of molecular H_2_O and its dissociation products. At elevated temperatures, a negative charge generated on the surface by a vigorous H_2_O/GaN interfacial chemistry induced an increase in both the surface work function and upward band bending. We tracked the dissociative adsorption of H_2_O onto the GaN(0001) surface by recording the core-level photoemission spectra and obtained the electronic and chemical properties at the H_2_O/GaN interface under *operando* conditions. Our results suggest a strong correlation between the electronic and chemical properties of the material surface, and we expect that their evolutions lead to significantly different properties at the electrolyte/electrode interface in a photoelectrochemical solar cell.

Photoelectrochemical (PEC) solar cells that produce hydrogen via water splitting offer a simple and efficient approach to the future sustainable supply of energy^1^,^2^,^3^,^4^,^5^. The performance of these solar cells is directly related to their electrolyte/photoelectrode interfacial properties; therefore, the selection of photoelectrode materials involves strict criteria including a reasonable band gap for the efficient capture of solar energy, proper aligment of the band edge with the oxidation-reduction potential of water, and sufficient chemical and electrical stability under harsh environmental conditions, such as low/high pH, oxidizing media, and high temperatures^6^,^7^,^8^. In particular, achieving tunable Fermi level and surface band bending properties remain universal challenges for solving issues such as the low efficiency and low stability of solar cells. As was demonstrated recently, this decreased efficiency is influenced by limitations in the transport of spatially-separated electron-hole pairs, which is usually governed by photoelectrode surface band bending^9^,^10^,^11^,^12^. For example, upward band bending in an n-type material directs the photo-excited electrons towards the bulk region, thereby creating an electron depletion layer at the surface that decreases the electron concentration significantly^11^,^12^,^13^.

III-N semiconductors possess many favorable properties that make them promising candidates as photocathode materials in future PEC solar cells. By varying the elemental stoichiometry or the application of co-catalysts to these semiconductors, various properties, such as band gap, lattice constant, and refractive index can be tuned precisely to satisfy a specific system, which also makes them electronically and architecturally flexible^1^,^14^,^15^,^16^. Because of the wide range of applications of GaN in optoelectronic devices, many studies have focused on its surface stability and manufacturing processing techniques^8^,^17^. Recently, considerable attention has also been focused on GaN as a material for photoelectrodes, thus, the H_2_O/GaN interfacial properties have been investigated both theoretically^9^,^10^,^18^ and experimentally^7^,^8^,^19^,^20^,^21^. Several research groups have reported that H_2_O dissociates onto the GaN surface at room temperature with an optimal coverage varying from 0.375 to 1 monolayer (ML)^8^,^18^,^19^,^20^,^22^ and a dissociation activation barrier ranging from thermadynamically favorable to 1.6 meV^7^,^8^,^19^,^21^, which is well below the thermal energy at room temperture (~25 meV)^5^. In addition, the intial investigations of the band structure of GaN under ultra-high vacuum (UHV) conditions, as performed by Bermudez *et al.*, indicated that there were detectable band bending and surface photovoltage (SPV) effects on a pristine surface and a surface exposed to H_2_O^22^,^23^,^24^. Since GaN is a promising candidate for photoelectrodes, an understanding of the interfacial chemistry and electronic properties of H_2_O/GaN under operational conditions is of particular importance in the improvemenet of the efficiency and lifetime of PEC solar cells for water splitting.

Recent advances in photoemission spectroscopic techniques, which have been demonstrated to be powerful tools for studying material surface properties under UHV conditions, have now enabled the study of these properties under ambient and operating (*operando*) conditions^25^,^26^,^27^. In this work, we used ambient pressure X-ray photoelectron spectroscopy (AP-XPS) to track the physicochemical processes that occur on the GaN(0001) surface over wide ranges of pressure and temperature, thus approaching the operational conditions of PEC cells. In general, the compositional, structural, and electronic properties of a material surface under ambient conditions differ substantially from those under UHV conditions due to changes in the surface free energies, which can vary by 0.3 eV or more^25^,^26^. By performing AP-XPS studies, we were able to follow the GaN surface chemistry at a molecular level and track the evolution of its surface electronic properties that govern the performances of PEC devices with respect to work function, band bending, SPV effect, ionization energy, and electron affinity under *operando* conditions.

A detailed description of the instrument can be found in our previous work^28^,^29^, and the specifics of the experimental procedure and corresponding analysis conducted in this work are included in the Supporting Information (SI). An undoped GaN(0001) wafer was cleaned by cycles of N_2_^+^ bombardment, followed by annealing in N_2_ (3 × 10^−7^ mbar) at ~1200 K for surface structure restoration^22^,^23^,^30^,^31^,^32^,^33^,^34^. This pre-treatrement resulted in a GaN (0001)-1 × 1 reconstruction, which we imaged using low energy electron diffraction (LEED). The photoemission spectra were collected across a range of H_2_O pressures from 10^−10^ to 5 mbar and temperatures from 298 to 773 K. Photoemission spectra for a few representative conditions, using two calibration methods for binding energy scales in order to identify chemical or electronic changes of GaN surface, are presented in Figs 1 and 2.

## Chemical properties

**Figure 1:**
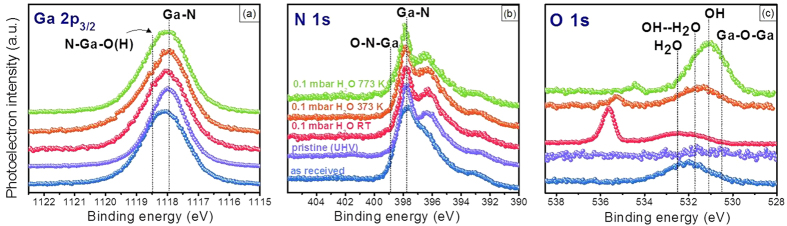
Photoemission spectra of Ga 2p_3/2_ (**a**), N 1 s (**b**), and O 1 s (**c**) obtained under several experimental conditions: as received, pristine (UHV), and 0.1 mbar of H_2_O at room temperature (RT), 373 K, and 773 K. The features at BEs below 397 eV can be attributed to Ga Auger peaks, e.g., L_2_M_45_M_45_ at ~396 eV. Notes: The binding energy scale was calibrated to the N 1 s of N-Ga bond at the BE of 397.8 eV. The Ga 3d spectra obtained for the same experimental conditions and using the same calibration method of binding energy scale are presented in Figure S1 for a better comparison of chemical changes.

We monitored H_2_O/GaN(0001) interfacial chemistry under various conditions from the photoemission spectra of Ga 2p_3/2_, N 1 s, and O 1 s (Fig. 1). At room temperature, H_2_O dissociative adsorption onto GaN(0001) was indicated by the appearance of a new component in the O 1 s spectra at 531.8 eV, which was attributed to the formation of OH-based surface products (Fig. 1c)^28^,^29^,^35^. In addition to the presence of H_2_O dissociative products, molecular water also existed, predominatly in the upper adsorption layers, as suggested by a slight broadening of the peak in the O 1 s spectra (Fig. 1c)^28^,^29^,^35^,^36^,^37^. We observed a strong correlation between the increase in the O 1 s intensity and the increase in H_2_O pressure, suggesting that the dissociation process was facilitated by more extensive water coverage. We also assigned a feature at the lower binding energy (BE) of the O 1 s spectra (530.6 eV) to the Ga-O-Ga species, which most likely formed as the result of a Ga-terminated surface structure. At higher temperatures, we observed more significant spectral changes. As shown in Fig. 1a, the presence of a much stronger extension of the Ga 2p_3/2_ signal at a higher BE suggested the formation of Ga oxides or hydroxides on the surface^29^. Correspondingly, a feature at 399 eV that appeared in the N 1 s spectra at 773 K indicated the formation of N-O bonds^38^. Based on the maximum shift of the O 1 s peak to ~531 eV, we believe that amorphous Ga-N-O structures also formed at the H_2_O/GaN interface. During initial molecular adsorption at room temperature, H_2_O with two lone pairs of electrons binds as a Lewis base (OH) to the empty orbital of Ga atoms (Lewis acid)^6^. Such a system is usually unstable and it dissociates to form Ga-OH and N-H bonds (Fig. 3). From point of view of electrostatic interactions, the Ga atom is positively charged and N is negatively charged in GaN, therefore partially positively charged H and partially negatively charged OH of initially molecularly adsorbed H_2_O covalently bind to N and Ga, respectively, preceded by H_2_O dissociation. The oxidation of Ga was observed in the Ga 2p and Ga 3d spectra, but N is highly electronegative, thus the formation of N-H bonds does not lead to apparent changes in the N 1 s photoemission spectra. However, there are recent theoretical studies which report the formation of N-H bonds for the H_2_O/GaN system^6^,^7^,^19^. Similar structures were observed for other III-V semiconductors, such as GaP, after exposure to H_2_O^29^. At elevated temperatures, the hydroxylation and oxidation of GaN were enhanced significantly, and a proposed reaction pathway will be discussed below. In contrast to other studies of H_2_O/III-V interfaces^28^,^29^,^39^, we observed no significant changes in the shapes of the core-level photomission spectra of Ga and N. The absence of such changes was due primarily to the following reasons: i) the formation of species such as Ga-OH and O-Ga-OH, which have spectral features that overlap with those of the Ga-N bond, and thus cannot be clearly distinguished, and/or ii) the Ga-N bond is much stronger than other III-V bonds, and thus GaN is chemically more stable.

## Electronic properties

Interestingly, in addition to the chemical changes demonstrated in the photoemission spectra, we observed that the interactions of H_2_O with GaN(0001) under different experimental conditions led to significant changes in the electronic properties at the H_2_O/GaN interface.

**Figure 2:**
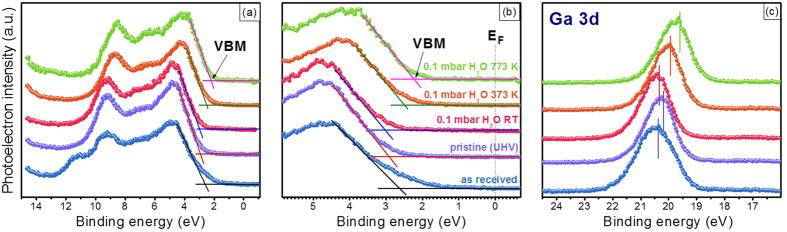
Photoemission spectra of the valence band (**a**), zoom in view of the valence band (**b**) and Ga 3d (**c**) under several experimental conditions: as received, pristine (UHV), and 0.1 mbar of H_2_O at RT, 373 K and 773 K. The perpendicular lines in (**c**) indicate a position of Ga 3d_5/2_ that originates from the Ga-N component and is determined by peak fitting of Ga 3d photoemission spectra (Figure S2). Note: The binding energy scale was calibrated to the Fermi edge of pristine Au(111).

**Figure 3:**
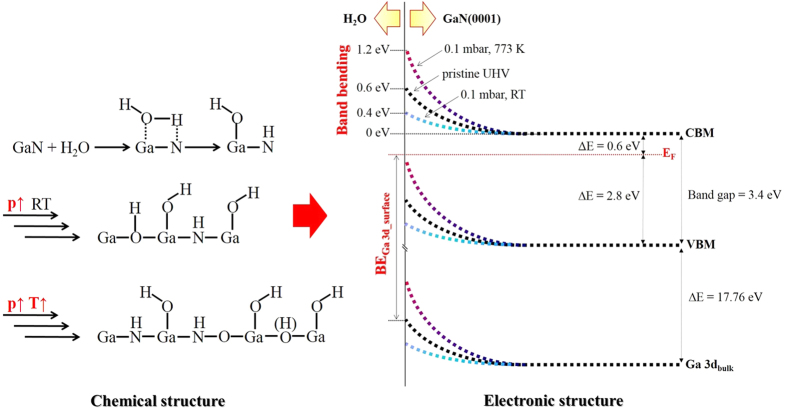
Scheme showing simplified chemical and band structure evolutions at the H_2_O/GaN(0001) interface. The band structures of three representative experimental conditions were used, i.e., UHV, 0.1 mbar of H_2_O at room temperature, and 0.1 mbar of H_2_O at 773 K.

Figure 2 shows the photoemission spectra of the valence bands and the Ga 3d core levels for several experimental conditions, i.e., as received, UHV, and 0.1 mbar H_2_O at 293, 373, and 773 K. The BE scale of these spectra was calibrated with zero BE corresponding to the Fermi level (E_F_) of a pristine Au (111) surface. The near-surface band structure of GaN(0001) was determined by measuring the BE difference of E_F_ with respect to the valence band maximum (VBM)^22^,^32^,^40^,^41^. The position of the VBM was measured by the linear extrapolation of the edge to the baseline of the spectra (Fig. 2). The E_F_-E_V_ was estimated to be 2.8 eV for the pristine GaN(0001) surface, which is consistent with previous reports for the Ga-terminated polar surface of an undoped GaN(0001) crystal^41^,^42^. For better visualization, Fig. 2b shows a zoom-in view of the valence bands. Peak positions in the valence band and the core-level photoemission spectra shifted consistently under different experimental conditions, and Table S1 summarizes their BE values. For the pristine GaN (0001) surface, the BE of the Ga 3d peak (BE Ga 3d_surface_) was measured at 20.15 eV with respect to E_F_ (Fig. 2 and Table S1). As shown in Fig. 3, using a value of 17.76 eV^22^,^43^ for the energy separation (ΔE(VBM-BE Ga 3d_bulk_)) between the VBM and core-level BE of Ga 3d_bulk_ and 3.4 eV for the band gap of GaN, an energy separation ΔE(CBM-E_F_) of 0.6 eV was obtained from E_F_ to the conduction band minimum (CBM). The energy separation between the CBM and BE Ga 3d_bulk_ remained constant before and after band bending. Therefore, we obtained the following relationship:


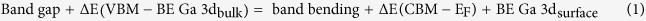


After inserting all the known values into the above equation, we used the following equation to calculate band bending:


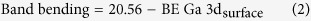


From these calculations, we estimated an apparent upward band bending of 0.39 eV for the pristine GaN(0001) surface. Using the same method, band bending values were obtained under various experimental conditions (Table S1).

However, the band bending obtained above was actually a combination of the real band bending and the SPV effect. Because of the existence of the vacuum/semiconductor interface, the surface of a semiconductor has a built-in space charge electric field, which is defined as a depletion layer^22^,^23^. X-ray excitation can cause the creation of electron-hole pairs, which can partially cancel out the original built-in depletion zone field in the GaN surface region and decrease surface band bending, leading to core-level peak shifts towards a higher BE. To determine the real contribution of band bending, the SPV effect should be eliminated, e.g., by increasing the temperature of the sample or by decreasing the X-ray power^22^,^23^,^44^,^45^. In this study, we observed a difference of ~0.2 eV in the BE of Ga 3d for the GaN surface before and after annealing at 773 K. Taking into account the correction for the SPV effect, the pristine GaN surface had ~0.6 eV of upward band bending and the real band bending values under various experimental conditions are listed in Table S1.

The extent of band bending is affected by exposing the surface to H_2_O vapor at room temperature. As shown in Fig. 2 and Table S1, at 293 K, the upward band bending of the GaN(0001) surface decreased by ~0.2 eV at an H_2_O pressure of 0.1 mbar. A similar effect was also reported by Lorenz *et al.*^18^ and Bermudez *et al.*^22^, in which, without consideration of the SPV effect, both groups observed flattened bands of the GaN surface after H_2_O exposure^18^,^22^. Such flattening of the band bending is most likely related to the removal of the dangling bonds that are present on the pristine GaN surface^46^,^47^. Interestingly, at elevated temperatures, the shifts in Ga 3d and the valence band spectra toward lower BEs (Fig. 2) were actually a combination of the removal of the SPV effect and the increase of upward band bending, due to the formation of oxide-related species at the H_2_O/GaN interface. This increase was caused by the formation of oxygen-induced negative charges on the GaN surface^23^. The formation of oxides was also confirmed in the O 1 s spectra, in which a high-intensity peak was observed at 531 eV (Fig. 1)^23^. Similarly, the Ga 2p_3/2_ and N 1 s spectra were broadened at higher BEs, suggesting the formation of Ga-O and N-O bonds^48^. As shown in Fig. 3, according to the analysis of the chemical and electronic properties given above, we were able to elucidate both a simplified mechanism of H_2_O dissociation and a configuration of the band structure at the H_2_O/GaN interface in an H_2_O environment. Generally, a pristine GaN(0001) surface exhibits upward band bending, and this bending effect is observed consistently in both valence band and core-level photoemission spectra. H_2_O dissociatively adsorbs onto the GaN surface at room temperature, resulting in decreased upward band bending, due to the removal of dangling bonds. However, at elevated temperatures, the H_2_O/GaN interfacial chemistry becomes more vigorous and causes the formation of surface oxides and/or hydroxides, which create a negativly charged surface and lead to greatly increased band bending. Our results indicate that the formation of oxides on a III-V photoelectrode surface can significantly influence the electrical properties of a PEC solar cell.

In addition to changes in band bending, other electronic properties of GaN were affected by the H_2_O/GaN interfacial chemistry, namely work function and electron affinity. We monitored the evolution of the work function of the GaN(0001) surface under *operando* conditions, by using Ar gas as a probe, and measured changes in the BE of Ar 2p near the surface region. A detailed description of the method employed for estimating the work function is provided in the SI^49^. The pristine GaN surface had a work function of 3.7 eV, and it decreased slightly upon H_2_O exposure, due to the electron donation from the upper layer physisorbed water to the H_2_O/GaN interface^49^. However, the reaction of H_2_O dissociation products, i.e., OH, H, and O, with the GaN surface under elevated temperatures increased the surface work function and reached 4.5 eV at an H_2_O pressure of 0.1 mbar and at 773 K. Recent studies indicated that the change in the work function in the III-V semiconductors was closely related to the changes in surface potential, which were associated with changes in the surface dipole moment^50^,^51^,^52^. Similarly, Williams *et al.*^50^ indicated that water dissociation products affected stronger a surface electronic structure than molecularly adsorbed H_2_O. The formation of Ga(N) oxide/hydroxide species, especially under high-temperature conditions, was most likely responsible for the change in work function. Electron affinity is defined as the difference in energy between the vacuum level and CBM, and it can vary drastically when surface dipoles form at the H_2_O/GaN interface.

We obtained the net change in electron affinity (Δχ) by the following equation^22^,^30^:


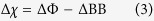


where ΔΦ is the change in work function, and ΔBB is the change in band bending. Therefore, the changes in electron affinity under various experimental conditions can be obtained (Table S1). Using an electron affinity of 3.8 eV as a reference value for the pristine GaN surface^41^, we were able to determine the electron affinities under different conditions (Table S1).

To summarize, we studied the chemical and electronic structures of the H_2_O/GaN(0001) interface using AP-XPS under several different conditions. H_2_O dissociated readily at room temperature and induced a flattening of the band bending at the interface. In contrast, at elevated temperatures, upward band bending increased considerably due to the removal of the SPV effect and the reaction of H_2_O dissociation products with GaN and the formation of Ga(N) oxides/hydroxides. Further, because of the changes in surface potential and dipole moment, other electronic properties were also affected by the H_2_O/GaN interfacial chemistry, including work function and electron affinity. The consistent correlation between the electronic and chemical properties at the H_2_O/GaN interface facilitated our understanding of the PEC process in GaN-based photoelectrodes. This work was a first attempt to use AP-XPS to investigate both the band structure and the surface chemistry of III-V semiconductors under *operando* conditions. We believe this approach will produce results that are even more meaningful when used for entire devices rather than just for photoelectrodes.

## Additional Information

**How to cite this article**: Zhang, X. and Ptasinska, S. Electronic and chemical structure of the H_2_O/GaN(0001) interface under ambient conditions. *Sci. Rep.* **6**, 24848; doi: 10.1038/srep24848 (2016).

## Supplementary Material

Supplementary Information
