# Supplementary material for: Electronic and chemical structure of the H2O/GaN(0001) interface under ambient conditions
Source: Sci Rep. 2016 Apr 25;6:24848. doi: 10.1038/srep24848 (PMC4843015; doi:10.1038/srep24848)
Supplement: Supplementary Information [file srep24848-s1.pdf]

# Electronic and chemical structure of the $H_2O$/GaN(0001) interface under ambient conditions

Xueqiang Zhang[1,2] and Sylwia Ptasinska[1,3]*

*[1]Radiation Laboratory, [2]Department of Chemistry and Biochemistry and [3]Department of Physics, University of Notre Dame, Notre Dame, IN 46556, USA*

Supporting Information

## I. Experimental Methods

All experiments were performed using the ambient pressure X-ray Photoelectron Spectroscopy (AP-XPS) system in the Notre Dame Radiation Laboratory. The photoemission spectra were obtained with a SPECS Phoibos 150 Hemispherical Energy Analyzer that was coupled to a differential pumping electrostatic lens system. Al Kα X-rays (1486.7 eV) were generated by a micro-focus X-ray (XR-MF) source. The X-rays were monochromatized and then transmitted through a silicon nitride membrane of a reaction cell that was installed in an analysis chamber. The reaction cell was custom-designed to conduct surface characterization at elevated pressures (up to 25 mbar) and temperatures (up to 873 K).

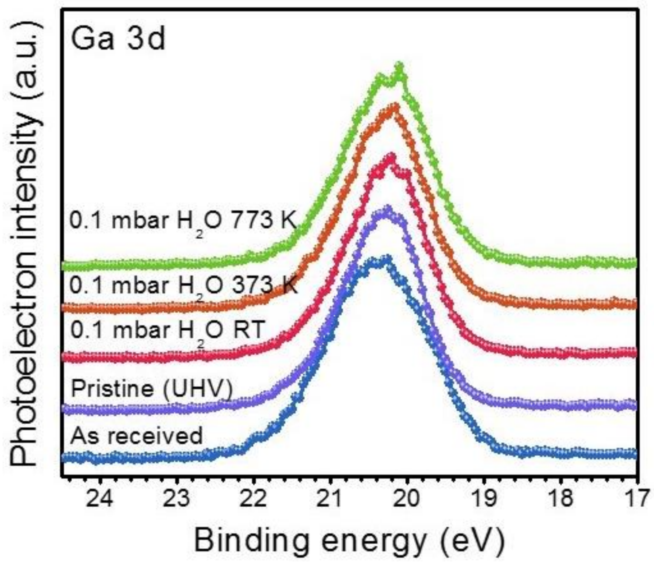


Figure S1. Photoemission spectra of Ga 3d obtained under several experimental conditions: as received, pristine (UHV), and 0.1 mbar of $H_2O$ at room temperature (RT), 373 K, and 773 K. Note: The binding energy scale was calibrated to the N 1s of N-Ga bond at the BE of 397.8 eV.

Photoemission spectra in the isothermal conditions were stabilized within ~10 min after introducing $H_2O$ vapor at particular pressures. The data acquisition time typically was about 0.5

h, and for higher $H_2O$ pressures (~5 mbar), up to 1 h. No noticeable time-dependent changes were observed in the photoemission spectra at room temperature. In isobaric conditions, the spectra were recorded after annealing the sample at a rate of 5 K/min, followed by a 20-min period for stabilization. We observed minor time-dependent variations in the intensities of spectral peaks (within 10%) in the isobaric experiments. However, such variations influenced the relative quantity of different chemical species formed at the $H_2O$/GaN interface, not the types of these species. The photoemission spectra of Ga 3d are shown in Figure S1.

## II. Surface pretreatment

An undoped GaN(0001) wafer was purchased from MTI, USA (Ga-face, Resistivity<0.5 Ohm-cm). The GaN(0001) wafer was cleaned in a preparation chamber of the AP-XPS system using a procedure reported by Burmudez et al.,[1-3] involving cycles of 0.5 keV $N_2^+$ bombardment at an angle of 45º with respect to the surface normal for contaminant removal, followed by annealing to 1173 K in a $N_2$ pressure of $3\times10^{-7}$ mbar for surface restoration.[4,5] After such pretreatments, we observed a sharp low energy electron diffraction (LEED) pattern that suggested the formation of a GaN(0001)-(1×1) reconstruction. Immediately after cleaning and recording LEED patterns in the preparation chamber, the GaN crystal was transferred into an adjacent XPS analysis chamber to confirm the absence of any surface contaminants. We monitored the crystal temperature using a K-type (chromel-alumel) thermocouple sandwiched between the GaN(0001) wafer and a molybdenum sample holder. Prior to measurement, the thermocouple was calibrated using a Lumasense Pyrometer. The photoemission spectra of C 1s were recorded frequently, and no carbon contamination was detected throughout the experimental procedures. Deionized water (Milli-Q, Millipore) was freeze-pump-thawed several times to remove gaseous contaminants prior to the introduction of vapor into the reaction cell.

Single crystals of Cu(111), Ag(111), Pt(111), and Au(111) were purchased from Goodfellow, USA, and they were cleaned using standard procedures before being introduced into the reaction cell as reference samples to create a standard working equation for the work function measurements. In order to clean these single metal crystals, cycles of $Ar^+$ bombardment were followed by annealing. The following parameters were used: 1 keV and 800 K (Cu(111)), 1 keV and 800 K (Ag(111)), 1.25 keV and 1100 K (Pt(111)), and 0.5 keV and 1100 K (Au(111)). Surface cleanliness also was monitored by XPS. No contaminants were observed within the detection limit in the C 1s, N 1s, or O 1s regions in the survey spectra. Argon gas (99.999% pure) was used to probe work function, and was used as received. A residual gas analyzer (RGA) connected to a differential pumping stage monitored the purity of the Ar and the $H_2O$ vapor in the reaction cell. No contaminants were measured above the detection limit of the RGA.

## III. Surface analysis

*Spectral analysis*: The photoemission energy scales were referenced to the Fermi edge of clean Au(111) and were recalibrated on a weekly basis. Samples were measured in the dark to minimize the surface photovoltage (SPV) effect. However, in order to exclude any band bending

effect, we used the Ga-N component with a binding energy (BE) of 397.8 eV,[6,7] for BE scale calibration. Therefore it is important to note that we used two methods for BE scale calibration, in which the BE scale was referred either to the N 1s of the N-Ga bond at 397.8 eV or to the Fermi edge of pristine Au(111). In order to identify the chemical shifts the calibration method using N 1s was performed, since chemical species were assigned based on known BE values.[6,7] However this method excludes the band bending effect, which is different from the chemical shifts and causes a shift of all peaks in the spectra. The analyses of the photoemission spectra were performed using CasaXPS software by fitting a Voigt function, which was obtained by convoluting Gaussian and Lorentzian functions, with a defined BE, full width at half maximum (FWHM), and a Gaussian:Lorentzian mixing ratio of 70:30. A flexibility of 0.1 - 0.2 eV for the BE and FWHM was used for peak fitting. The FWHM of the Au $4f_{7/2}$ peak was 0.5 eV with a pass energy of 20 eV and the inherent lifetime broadening of this peak has been reported to be ~0.3 eV.[8] The BEs of the Ga-N component in Figure 2c were obtained by fitting the Ga 3d spectra with multiple components, including spin-orbital splitting and formation of oxides and hydroxyls as shown in Figure S2.

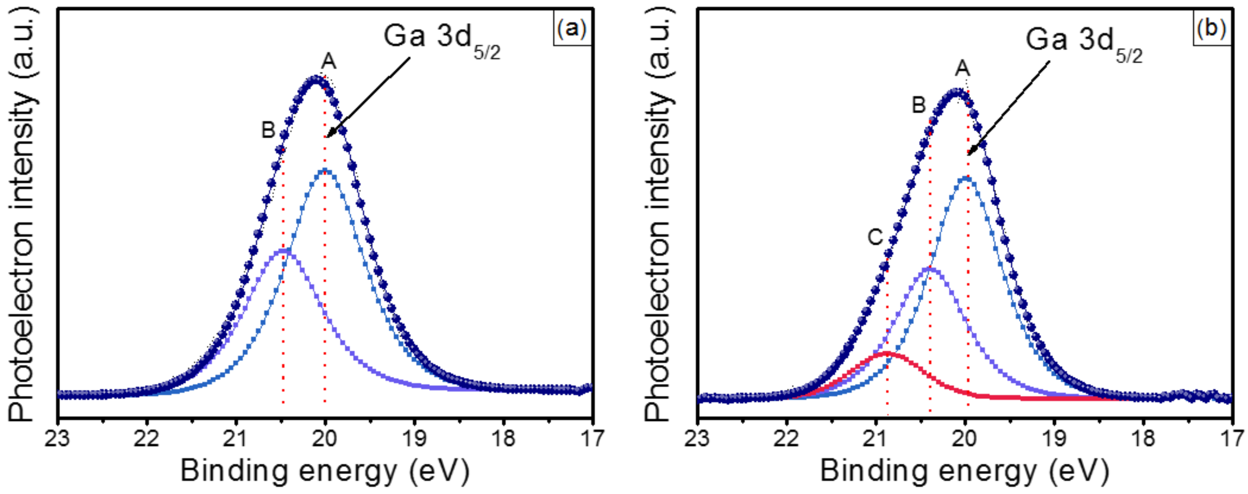


Figure S2. Peak fitting of the Ga 3d photoemission spectra under two representative conditions: a) pristine (UHV) and b) 0.1 mbar of $H_2O$ at 773 K. A and B represent Ga $3d_{5/2}$ and Ga $3d_{3/2}$ due to spin-orbital splitting for the Ga-N component, respectively, and C represents Ga oxidation/hydroxylation without identifying specific surface species. Note: The binding energy scale was calibrated to the N 1s of N-Ga bond at the BE of 397.8 eV.

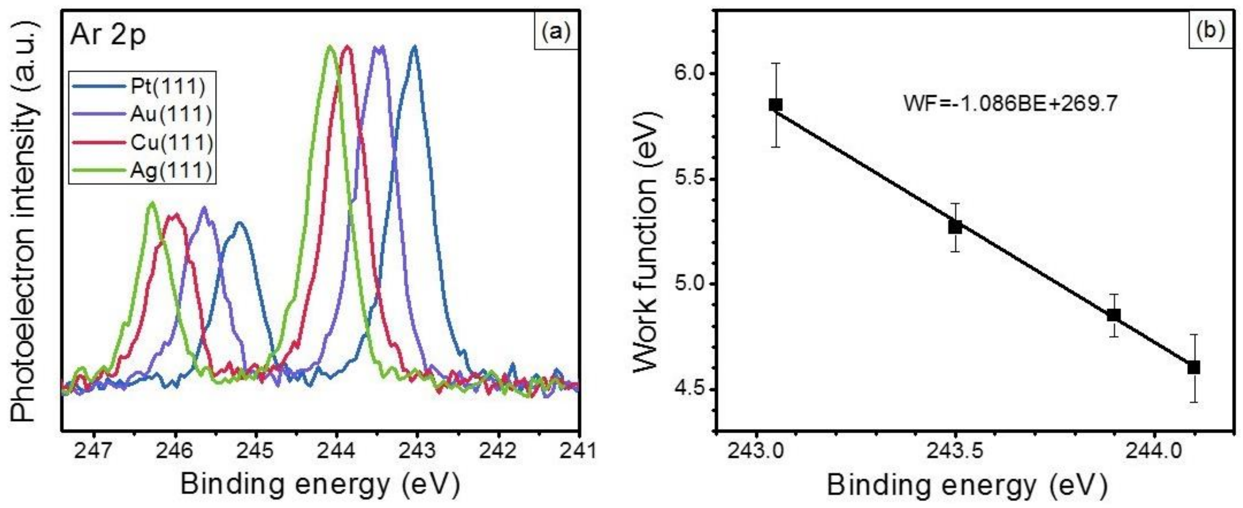


Figure S3. a) Photoemission spectra of near-surface gas-phase Ar at a pressure of 0.5 mbar in the reaction cell obtained for the Pt(111), Au(111), Cu(111), and Ag(111) single crystals; b) Linear fitting of the relationship between the Ar $2p_{3/2}$ binding energy and work function of Pt(111), Au(111), Cu(111), and Ag(111) single crystals.

*Estimates of work function*: Changes in surface work functions were determined from the photoemission spectra of gas-phase Ar at the near-surface region of the GaN(0001) wafer. This method has been used previously for liquid surfaces[9,10] and nanoparticles.[11,12] In this study, we estimated the work functions of GaN(0001) from BE shifts in the Ar $2p_{3/2}$ spectra recorded in the reaction cell at an Ar pressure of 0.5 mbar. The changes in the BE of the near-surface Ar atoms were correlated to the work function of the material surface.[11,12] By using reference samples for which the work functions are well-known, the BE of Ar can be converted directly into the work function of the GaN(0001) surface. Single crystals of Cu, Ag, Pt, and Au with (111) orientations have been reported to have work functions of 4.85 ± 0.10,[13-15] 4.60 ± 0.16,[15-18] 5.90 ± 0.20,[11,19,20] and 5.27 ± 0.12 eV,[11,15,18,21-26] respectively. By plotting these values as a function of the BE of the Ar $2p_{3/2}$ peak (Figure S3), we obtained the following linear dependence:

$$WF = -1.086\, BE + 269.7 \qquad \text{(Equation S1)}$$

where WF is the work function of a particular sample, and BE is the BE of the Ar $2p_{3/2}$ peak.

Therefore, the work function of the GaN(0001) wafer under various experimental conditions could be estimated by the introduction of Ar at a pressure of 0.5 mbar, while $H_2O$ was still present on the GaN surface. The Ar 2p spectra for samples treated under several experimental conditions were recorded, after which the BEs of Ar $2p_{3/2}$ were extracted. The changes in the work function were determined using Equation S1, and they are listed in Table S1.

Table S1. Measured and estimated values of band bending (BB), work function (WF), electron affinity (EA), ionization energy (IE), and their relative changes under different experimental conditions with respect to pristine GaN. Note: The values were obtained from peak fittings of photoemission spectra and do not necessarily indicate the accuracy of the spectrometer.

| **Energy (eV)** | **As received** | **Pristine (UHV)** | **0.005 mbar, RT** | **5 mbar, RT** | **0.1 mbar, 373 K** | **0.1 mbar, 773 K** |
|---|---|---|---|---|---|---|
| **VBM** | 2.60 | 2.87 | 3.05 | 3.08 | 2.49 | 2.20 |
| **Ga 3d** | 20.40 | 20.15 | 20.33 | 20.37 | 19.96 | 19.58 |
| **BB** | 0.16 | 0.41 | 0.23 | 0.19 | 0.60 | 0.98 |
| **SPV correlated BB** | 0.36 | 0.61 | 0.43 | 0.39 | 0.80 | 1.18 |
| **ΔBB ( ±0.1 eV)** | -0.25 | 0 | -0.18 | -0.22 | 0.19 | 0.57 |
| **ΔEA ( ±0.2 eV)** | 0.03 | 0 | 0.11 | 0.06 | 0.03 | 0.23 |
| **EA** | 3.83 | 3.80 | 3.91 | 3.86 | 3.83 | 4.03 |
| **ΔWF (±0.1 eV)** | -0.22 | 0 | -0.07 | -0.16 | 0.22 | 0.80 |
| **IE** | 7.22 | 7.20 | 7.31 | 7.26 | 7.23 | 7.43 |
| **SPV** | ~0.2 | | | | | |

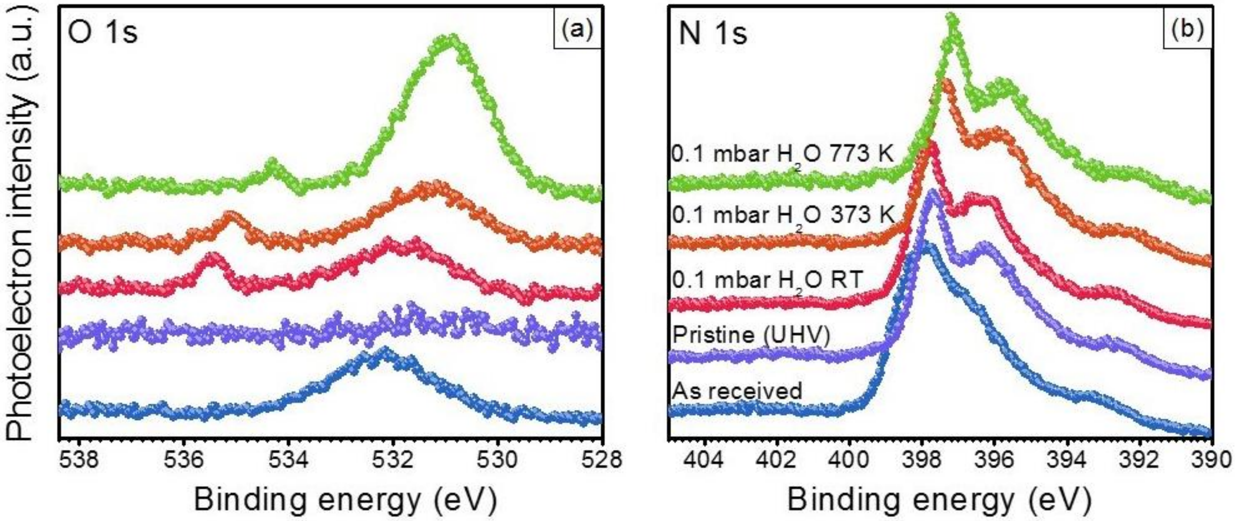


Figure S4. Photoemission spectra of O 1s (a) and N 1s (b) under several experimental conditions: as received, pristine (UHV), and 0.1 mbar of $H_2O$ at RT, 373 K, and 773 K. Notes: The binding energy scale was calibrated to the Fermi edge of pristine Au(111).

*Electronic properties*: Using the method described in the main text, we were able to determine the changes in band bending, work function, and electron affinity under different experimental conditions (summarized in Table S1). Figure S4 shows the photoemission spectra of O 1s and N 1s, involving band bending and SPV effects under a few chosen experimental conditions that were used to estimate the electronic properties. The evolution of band bending, work function, and electron affinity are plotted and shown in Figure S5.

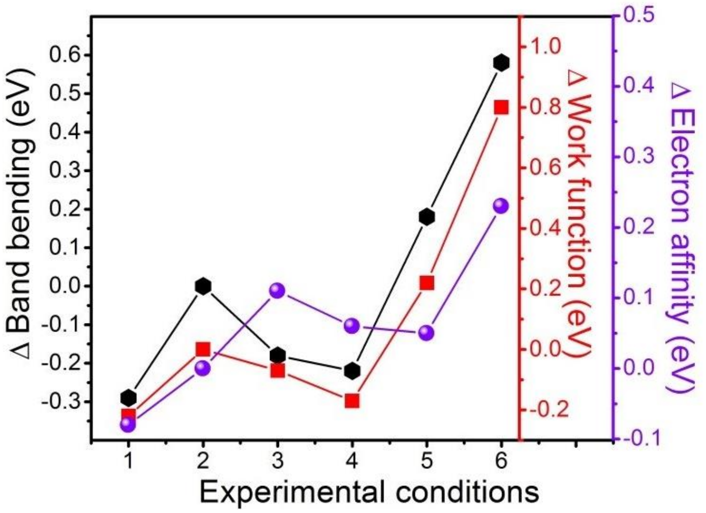


Figure S5. Evolutions of the changes in band bending, work function and electron affinity under different experimental conditions: as received (1), pristine (UHV) (2), 0.005 mbar $H_2O$ at room temperature (3), 5 mbar $H_2O$ at room temperature (4), 0.1 mbar $H_2O$ at 373 K (5), and 0.1 mbar $H_2O$ at 773 K (6).

## References


(1) Bermudez, V. M. & Long, J. P., Chemisorption of $H_2O$ on GaN(0001). *Surf. Sci.* **450**, 98-105 (2000).

(2) Bermudez, V. M., Koleske, D. D. & Wickenden, A. E., The dependence of the structure and electronic properties of wurtzite GaN surfaces on the method of preparation. *Appl. Surf. Sci.* **126**, 69-82 (1998).

(3) Long, J. P. & Bermudez, V. M., Band bending and photoemission-induced surface photovoltages on clean n- and p-GaN(0001) surfaces. *Phys. Rev. B* **66**, 121308 (2002).

(4) Oliver, R. A. et al., Gallium nitride surface preparation optimised using *in-situ* scanning tunnelling microscopy. *Appl. Surf. Sci.* **214**, 1-10 (2003).

(5) King, S. W. et al., Cleaning of AlN and GaN surfaces. *J. Appl. Phys.* **84**, 5248-5260 (1998).

(6) Yang, J., Eller, B. S. & Nemanich, R. J., Surface band bending and band alignment of plasma enhanced atomic layer deposited dielectrics on Ga- and N-face gallium nitride. *J. Appl. Phys.* **116** (2014).

(7) Wang, S., Zhang, X., Feng, Z. C. & Cui, Y., Surface chemical and local electronic properties of $Al_xGa_{1-x}N$ epi-layers grown by MOCVD. *Opt. Exp.* **22**, 17440-17447 (2014).

(8) Patanen, M. et al., Free atom 4f photoelectron spectra of Au, Pb, and Bi. *J. Electron. Spectrosc. Relat. Phenom.* **183**, 59-63 (2011).

(9) Siegbahn, H. & Lundholm, M., A method of depressing gaseous-phase electron lines in liquid-phase ESCA spectra. *J. Electron. Spectrosc. Relat. Phenom.* **28**, 135-138 (1982).

(10) Siegbahn, H., Electron-spectroscopy for chemical-analysis of liquids and solutions. *J. Phys. Chem.* **89**, 897-909 (1985).

(11) Axnanda, S. et al., Direct work function measurement by gas phase photoelectron spectroscopy and its application on PbS nanoparticles. *Nano Lett.* **13**, 6176-6182 (2013).

(12) Crumlin, E. J., Bluhm, H. & Liu, Z., *In-situ* investigation of electrochemical devices using ambient pressure photoelectron spectroscopy. *J. Electron. Spectrosc. Relat. Phenom.* **190**, 84-92 (2013).
(13) Gartland, P. O., Berge, S. & Slagsvol. B. J., Photoelectric work function of a copper single-crystal for (100), (110), (111), and (112) faces. *Phys. Rev. Lett.* **28**, 738 (1972).
(14) Takeuchi, K., Suda, A. & Ushioda, S., Local variation of the work function of Cu(111) surface deduced from the low energy photoemission spectra. *Surf. Sci.* **489**, 100-106 (2001).
(15) Duhm, S. et al., PTCDA on Au(111), Ag(111) and Cu(111): Correlation of interface charge transfer to bonding distance. *Org. Electron.* **9**, 111-118 (2008).
(16) Chelvayohan, M. & Mee, C. H. B., Work function measurements on (110), (100) and (111) surfaces of silver. *J. Phys. C: Solid State Phys.***15**, 2305-2312 (1982).
(17) Dweydari, A. W. & Mee, C. H. B., Work function measurements on (100) and (110) surfaces of silver. *Physica Status Solidi A-Appl. Res.* **27**, 223-230 (1975).
(18) Otalvaro, D., Veening, T. & Brocks, G., Self-assembled monolayer induced Au(111) and Ag(111) reconstructions: Work functions and interface dipole formation. *J. Phys. Chem. C* **116**, 7826-7837 (2012).
(19) Nieuwenh.B. E. & Sachtler, W. M., Crystal face specificity of nitrogen adsorption on a platinum field-emission tip. *Surf. Sci.* **34**, 317-336 (1973).
(20) Nieuwenh. B. E., Meijer, D. T. & Sachtler, W. M., Adsorption of xenon on platinum studied by field-emission microscopy. *Physica Status Solidi A-Appl. Res.* **24**, 115-122 (1974).
(21) Iijima, Y. et al., Oxygen reduction reaction activities of Pt/Au(111) surfaces prepared by molecular beam epitaxy. *J. Electroanalytical Chem.* **685**, 79-85 (2012).
(22) Sachtler, W. M. H., Dorgelo, G. J. H. & Holscher, A. A., The work function of gold. *Surf. Sci.* **5**, 221-229 (1966).
(23) Pescia, D. & Meier, F., Spin polarized photoemission from gold using circularly polarized-light. *Surf. Sci.* **117**, 302-309 (1982).
(24) Lecoeur, J., Bellier, J. P. & Koehler, C., Comparison of crystallographic anisotropy effects on potential of zero charge and electronic work function for gold (111), (311), (110) and (210) orientations. *Electrochim. Acta* **35**, 1383-1392 (1990).
(25) Tzeng, C. T., Lo, W. S., Yuh, J. Y., Chu, R. Y. & Tsuei, K. D., Photoemission, near-edge X-ray absorption spectroscopy, and low-energy electron-diffraction study of C-60 on Au(111) surfaces. *Phys. Rev. B* **61**, 2263-2272 (2000).
(26) Hagen, S., Leyssner, F., Nandi, D., Wolf, M. & Tegeder, P., Reversible switching of tetra-tert-butyl-azobenzene on a Au(111) surface induced by light and thermal activation. *Chem. Phys. Lett.* **444**, 85-90 (2007).
